# A Mini-Review on Gene Therapy in Glaucoma and Future Directions

**DOI:** 10.3390/ijms252011019

**Published:** 2024-10-14

**Authors:** Nicoleta Anton, Aida Geamănu, Raluca Iancu, Ruxandra Angela Pîrvulescu, Alina Popa-Cherecheanu, Ramona Ileana Barac, Geanina Bandol, Camelia Margareta Bogdănici

**Affiliations:** 1 Department of Ophtalmology, Grigore T. Popa University of Medicine and Pharmacy, 700115 Iaşi, Romania; anton.nicoleta1@umfiasi.ro (N.A.); camelia.bogdanici@umfiasi.ro (C.M.B.); 2 Ophthalmology Clinic, Sf. Spiridon Emergency Clinical Hospital, 700111 Iaşi, Romania; 3 Department of Ophtalmology, Carol Davila University of Medicine and Pharmacy, 050474 Bucharest, Romania; aida.geamanu@umfcd.ro (A.G.); alina_cherecheanu@yahoo.com (A.P.-C.); ramona.barac@umfcd.ro (R.I.B.); 4 Ears Nose Throat (ENT) Department, Grigore T. Popa University of Medicine and Pharmacy, 700115 Iaşi, Romania; geanina-bandol@umfiasi.ro

**Keywords:** glaucoma, gene therapy, gene mutations, intraocular pressure, retinal ganglion cells

## Abstract

Glaucoma is a group of optic neuropathies characterized by the degeneration of retinal ganglion cells and the loss of their axons in the optic nerve. The only approved therapies for the treatment of glaucoma are topical medications and surgical procedures aimed at lowering intraocular pressure. Gene therapy involves the insertion, removal, or modification of genetic material within cells to repair or compensate for the loss of a gene’s function. It describes a process or technology that enables the genetic modification of cells to produce a therapeutic effect. However, changing the genetic material alone does not extend the duration of overexpression of proteins that combat disease, nor does it facilitate the production of new proteins for this purpose. We reviewed the literature concerning the use of gene therapy in the treatment of glaucoma and explored the future directions that this innovation may offer. Three genes associated with glaucoma have been identified within these loci: myocilin/trabecular meshwork glucocorticoid response (TIGR) (GLC1A), optineurin (GLC1E), and WDR36 (GLC1G). Among these, the most extensively studied glaucoma gene is myocilin (a TM-inducible glucocorticoid response gene). Building on previous successes, researchers have begun to apply genetic therapeutic approaches to alleviate or reduce symptoms associated with ocular hypertension (OHT) and glaucoma-like optic neuropathy (GON). It is evident that several therapeutic strategies exist that modulate aqueous humor production and flow, thereby regulating intraocular pressure (IOP) and protecting retinal ganglion cells (RGCs) from apoptosis. With the emergence of gene therapy as a potentially viable approach to preserving vision, new methods for managing glaucoma may soon become available. Genomic therapy is a promising treatment option for glaucoma patients and has significant potential for widespread clinical application.

## 1. Introduction

Glaucoma is one of the leading causes of blindness worldwide and is influenced by both genetic and environmental factors. Many of these conditions are associated with elevated intraocular pressure (IOP), which is the most well-known risk factor, but not the only one, contributing to the development and progression of glaucomatous damage. The common pathological outcome is the death of retinal ganglion cells (RGCs), characterized by damage to the optic nerve head, followed by the apoptotic death of these cells. Traditional models of glaucoma treatment have primarily focused on reducing intraocular pressure, as it is the only factor known to lower the risk of progression in most patients. However, currently available treatments, such as the daily use of eye drops, often result in poor patient compliance, particularly among the elderly. Patients may also experience allergic reactions to topical medications or find it difficult to adhere to a consistent treatment regimen, leading to the exploration of alternative therapeutic approaches. DNA encodes information that directs the production of proteins, which are essential for our bodies to function. Mutations in genes can either be inherited or develop over time, resulting in abnormal proteins that may adversely affect our health [1,2,3,4,5]. Studies have shown that gene therapy is emerging as a potentially viable option for preserving vision, offering new avenues for managing glaucoma. However, it remains a less commonly known technology, partly because it is not widely utilized due to the limited number of patients eligible for such treatment and partly because it is a relatively under-researched field [3]. Researchers are exploring the use of gene-based therapies that can be more effective than conventional medications and require less frequent administration, potentially as little as once every 1 or 2 years. In cases of hereditary glaucoma caused by recessive or dominant genes, gene replacement and gene silencing strategies are being investigated to counteract the detrimental effects of these mutations [6]. This paper aims to identify studies that utilize gene therapies in the treatment of glaucoma and to highlight the potential future directions of this innovative approach.

### A Brief History of Gene Therapy

**Figure 1:**
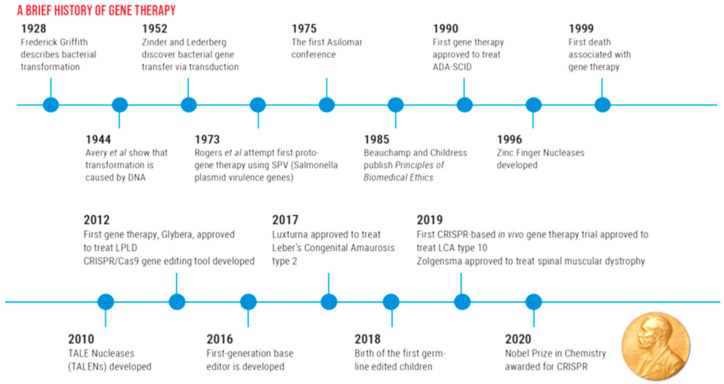
A brief history of gene therapy [8,9,10].

The concept of gene therapy was first introduced in 1928 when Frederick Griffith described bacterial transformation. Scientific advancements eventually culminated in the development of the first FDA-approved gene therapy, LUXTURNA, in 2017, for the treatment of Leber congenital amaurosis. The introduction and application of gene-editing technology, specifically CRISPR (Clustered Regularly Interspaced Short Palindromic Repeats) in 2019, marked a significant milestone for in vivo gene therapy. This breakthrough technology was awarded the Nobel Prize in Chemistry in 2020 (See Figure 1) [3,7].

Gene therapy can be used to treat human diseases through the following approaches [3,7]:

1. Gene replacement: Jean Bennett, MD, PhD, is a pioneer of this approach. She developed Luxturna, the first gene therapy approved for the treatment of Leber congenital amaurosis (LCA), a rare inherited eye condition.
2. Gene silencing: This method utilizes messenger RNA (mRNA) to inhibit the production of a specific protein.
3. Gene editing: This approach involves the use of CRISPR technology, which has gained significant attention recently. CRISPR stands for “Clustered Regularly Interspaced Short Palindromic Repeats”. The technique uses a guide RNA to precisely modify a gene within the patient’s genome.
4. Gene addition: This technique promotes the overexpression of a gene that can have a positive effect on disease outcomes. It is particularly useful when a protein is already being produced, but higher levels are needed to ensure cell survival or prevent disease progression.

Methods of introducing genetic material into cells: Genetic material is delivered using vectors—carrier particles that transport the new genetic information into target cells. There are primarily two types of vectors [3,7]:

- Viral vectors: These vectors can include RNA viruses, such as retroviruses, or DNA viruses, such as adenoviruses and adeno-associated viruses. Viral vectors introduce genetic material into cells in a manner similar to how standard viruses infect cells.
- Non-viral vectors: These can include DNA-related materials such as liposomes, “naked” DNA, or simple proteins. This approach is also known as “cell-based therapy”.

To date, six delivery systems have been tested for their ability to deliver genes to specific tissues or cells. These include adenoviruses (Ads), adeno-associated viruses (AAV), herpes simplex viruses (HSV), and lentiviruses (LV); such as feline immunodeficiency virus [FIV] and human viruses), as summarized in Table 1 [4].

Retroviruses, one of the viral vector options, are a type of RNA virus capable of converting their RNA into DNA. Some retroviruses can reverse-transcribe RNA into DNA and even integrate that DNA into the host genome. Once the genetic material is delivered to the target cells, the vector can interact with the host’s genetic material in various ways. One consideration is whether the delivered gene should be activated in every cell in the body (“ubiquitous” delivery) or only in specific cells (“cell-specific” delivery). This is regulated by what is known as the promoter sequence—a segment of genetic information within the vector. The promoter can act as a “master key”, initiating gene expression in all cells, or as a “specific key”, activating the gene only in a particular cell type. This mechanism allows for precise control over which cells ultimately produce the desired protein [3,7].

Luxturna (voretigene neparvovec-rzyl) is currently the only gene therapy in ophthalmology approved by both the FDA and the EU for the treatment of patients with Leber congenital amaurosis caused by mutations in the RPE65 gene. Jean Bennett, MD, PhD, is a pioneer in the development of this therapy and is considered the creator of Luxturna. The mutant RPE65 gene is just one of over 220 genes that, when mutated, can lead to inherited retinal dystrophies, accounting for approximately 2% of these cases. The most recent success in ocular gene therapy involves the treatment of retinitis pigmentosa (RP) through the insertion of a functional RPE65 gene using an AAV-based (adeno-associated virus) system to replace the non-functional gene. The RPE65 protein is essential for normal vision, and its replacement through gene therapy has shown promising results in restoring visual function [11,12]. Voretigene neparvovec-rzyl-AAV2 (Luxturna; Spark Therapeutics) was administered through bilateral subretinal injections to patients with retinitis pigmentosa (RP). Over time, vision improved in 27 out of 29 patients after at least one year of follow-up. The newly introduced gene produces a functional protein that replaces the defective one, thereby enhancing the visual cycle and enabling photoreceptor cells in the retina to respond more effectively to light. Studies suggest that patients with inherited RPE65 mutations in both maternal and paternal genes must have viable retinal cells for the treatment to be effective. Widely described by the media as a “vision-restoring” curative treatment, Luxturna was priced at USD 850,000 per patient. However, while voretigene neparvovec-rzyl represents a significant therapeutic advancement, most reports did not fully convey the study outcomes as documented by FDA reviewers. Evidence indicates that Luxturna does not restore normal vision but leads to only modest improvements, which may not be sustained long-term. Furthermore, in most cases, the assessment of visual function was deprioritized as a primary objective after mixed results were observed, with two patients experiencing permanent vision loss [13,14,15].

## 2. Results

We analyzed the literature on the use of gene therapy in the treatment of glaucoma and explored the potential future directions this innovation may offer. The review included studies, case reports, and reviews published in English from 2015 to 2023. All sources were obtained by searching relevant databases. This manuscript adhered to the procedures previously described by Green et al. [16]. We identified a total of 2284 articles related to gene therapy in glaucoma, including both review and research papers (ScienceDirect: 452, Web of Science: 790, Scopus: 823, and PubMed: 185). After applying the inclusion criteria—articles and reviews in English, published between 2015 and 2023, specifically focused on gene therapy in glaucoma, open access, and from reputable medical publications—a total of 643 studies were selected for further examination.

### 2.1. Gene Therapy in Intraocular Hypertension and Glaucoma Treatment

Deciphering the genetic codes for thousands of proteins involved in various bodily functions has been a monumental achievement for humanity. Ocular genetic studies have significantly contributed to our understanding of the pathological links between specific gene defects and protein dysfunctions. Several loci on the human genome have been identified as playing a role in the pathogenesis of glaucoma. Gene therapy refers to a process or technology that modifies cells genetically to produce a therapeutic effect. This modification can be performed in vitro in isolated cells, which are subsequently introduced into the body, or in vivo, directly within the organism. Gene therapy involves the delivery of DNA or RNA into cells using vectors, enabling these cells to generate the corresponding proteins encoded by the inserted genetic material. In Table 1, we present an overview of the types of tissues, genes, and vectors used in gene therapy [6,11,15,17,18].

Table 2 presents the genes relevant to the treatment of glaucoma. Studies have shown that an adenovirus (Ad) vector carrying the stromelysin metalloproteinase gene can be successfully transduced into trabecular meshwork (TM) cells in rats following intracameral injection. Efforts to study the reduction of elevated intraocular pressure (IOP) by modulating TM resistance have been limited by the lack of an appropriate animal model that does not involve scarring of this tissue. Monitoring IOP reduction in normotensive animals is more challenging than assessing IOP changes in hypertensive ones. Gene transfer to the TM offers a unique opportunity to create such a model by introducing genes that increase, rather than decrease, IOP [6]. The most extensively studied glaucoma gene with high penetrance is MYOC (myocilin), a TM-inducible glucocorticoid response gene. There are over 70 known MYOC mutations, all associated with elevated intraocular pressure (IOP) ranging from mild to severe. Some mutations, such as Gln368X, are commonly linked to adult-onset primary open-angle glaucoma (POAG), while others, such as Pro370Leu, are almost exclusively associated with early-onset glaucoma. Since myocilin mutations are autosomal dominant and likely cause disease through a gain-of-function mechanism, a gene therapy approach for patients with myocilin mutations would involve a combination of gene deletion and gene addition. This strategy would use siRNA to block the mRNA of the mutant allele while simultaneously introducing an overexpressed normal gene to increase the levels of a functional allele. Young patients with significantly elevated IOP, who are at high risk of severe vision loss and face a lifelong burden, could be strong candidates for this form of treatment [4].

Three genes associated with glaucoma have been identified within specific loci, including myocilin/TIGR (GLC1A), optineurin (GLC1E), and WDR36 (GLC1G). Myocilin (MYOC) was identified by Stone and colleagues in the GLC1A locus, which was the first reported locus for primary open-angle glaucoma (POAG) located on chromosome 1. In adult POAG populations, the prevalence of myocilin mutations ranges between 3% and 5%, making it the most common form of inherited glaucoma currently known. Myocilin-associated glaucoma is transmitted as an autosomal dominant trait [4,19,20].

OPTN is located in the GLC1E locus on chromosome 10. The phenotype of affected individuals with OPTN variants is notable for glaucoma associated with normal intraocular pressure (IOP), commonly referred to as normal tension glaucoma (NTG), in a large proportion of affected family members. The original report identified OPTN variants in over 16% of families with open-angle glaucoma [19].

WDR36 (WD40-repeat 36) gene variants were first reported to cause primary open-angle glaucoma (POAG) in 2005. The prevalence of WDR36 sequence variations is estimated to range between 1.6% and 17% in patients with POAG [19].

In addition to the genes mentioned above, over 20 gene variants have been associated with primary open-angle glaucoma (POAG), as summarized in Table 3. These include apolipoprotein E (APOE), optic atrophy 1 (OPA1), tumor protein p53 (TP53), tumor necrosis factor (TNF), interleukin-1 (IL-1), and cytochrome P450 1B1 (CYP1B1). CYP1B1 has been reported to be associated with early-onset POAG in Spanish, French, and Indian populations [20].

Variants of OPA1 have been associated with normal-tension glaucoma in Japanese and Caucasian populations. However, OPA1 variants are not linked to glaucoma in Caucasian, African American, or West African primary open-angle glaucoma (POAG) cases with elevated IOP (Liu et al., 2007) [21]. Most gene associations with primary open-angle glaucoma (POAG) have not been consistently validated by other researchers or across different populations [21,22]. The table below presents the types of genes associated with glaucoma.

Building on previous successes, researchers have started applying genetic therapeutic approaches to alleviate or reduce symptoms associated with ocular hypertension (OHT) and glaucoma-like optic neuropathy (GON). This strategy involves generating short interfering RNA (siRNA) oligonucleotides and delivering them to target cells using adeno-associated virus (AAV) vectors. One example includes the use of siRNA targeting the beta-adrenergic receptor in the ciliary body to mimic the effect of beta-blockers, thereby reducing aqueous humor (AQH) production and lowering intraocular pressure (IOP) in animal models with elevated IOP and in OHT/GON subjects [23,24]. Enhancing the outflow of aqueous humor from the anterior chamber of the eye to lower intraocular pressure (IOP) through genetic approaches, along with the direct protection of retinal ganglion cells and their axons, which form part of the optic nerve, as well as preserving retinal connections to the brain centers through drugs and/or gene therapy, represents new frontiers in ocular medicine [11].

With the advent and application of gene-editing technology, the CRISPR-Cas9 (Clustered Regularly Interspaced Short Palindromic Repeats) RNA-guided system has enabled the editing of mutant myocilin, which accumulates in and around the trabecular meshwork in some glaucoma patients, with the potential to lower intraocular pressure (IOP). This system has also been used to modify the aquaporin-1 gene in the ciliary body to reduce aqueous humor (AQH) production, thereby lowering IOP to treat ocular hypertension (OHT) and glaucoma-like optic neuropathy (GON) [2]. In treated eyes, IOP decreased to an average of 10.4 ± 2.4 mmHg compared to 13.2 ± 2.0 mmHg in the control group and 13.1 ± 2.8 mmHg in untreated eyes (*p* < 0.001; n = 12). In the study, wild-type C57BL/6J mice received a unilateral intravitreal injection of the combined vector mix, and after 3 weeks, the ciliary body was isolated for analysis. Results showed an average IOP reduction of 2.9 mmHg compared to the contralateral (untreated) eye, representing a decrease of approximately 22%. This level of reduction would be clinically significant if translated to glaucoma therapy, as studies have shown that even a 25% decrease in IOP can preserve vision [2,23,24,25].

Currently, several clinical trials are underway using gene therapy for the treatment of glaucoma and Leber’s optic neuropathy, which are summarized in Table 4. Ocular gene therapy trials account for 1.8% of all gene therapy trials worldwide (34 out of 2356) [26].

### 2.2. Gene Therapy for Neuroprotection in Glaucoma

In cases of glaucoma with medically controlled or normal IOP, the decline in visual function may continue to progress. It is believed that gene therapy could help protect against such losses. Consequently, several treatment strategies have been shown to effectively prevent retinal ganglion cell (RGC) death in vitro and in vivo through the administration of neuroprotective agents. Gene therapy for retinal protection has primarily focused on the viral vector-mediated delivery of neurotrophins, such as brain-derived neurotrophic factor (BDNF) and its receptor, both of which are depleted in glaucomatous optic neuropathy. RGC apoptosis in glaucoma involves a cascade of pro-apoptotic factors, including BAX, a member of the BCL-2 family of endogenous transcription factors that regulate the delicate balance between cell survival and death. Donahue et al. [27] developed an mCherry-BCLXL fusion protein that prevented the recruitment, translocation, and activation of BAX in mitochondria within cells treated with staurosporine, an apoptotic agent. This fusion protein was packaged into an AAV2 vector and used to transduce retinal ganglion cells (RGCs) through intravitreal injection, enhancing its overexpression. The transduced RGCs expressed mCherry-BCLXL in their soma and axons throughout the entire optic tract. Using an acute optic nerve injury model in mice, the transgene prevented the recruitment of a GFP-BAX fusion protein to mitochondria and provided long-term somal protection for up to 3 months after injury. The effectiveness of this gene therapy was further tested in an animal model of glaucoma, with mice receiving the treatment at 5 months of age, when glaucomatous changes typically begin. The results indicated that while mCherry-BCLXL gene therapy did not alter the long-term course of IOP elevation compared to untreated control mice, it significantly reduced RGC soma pathology and axonal degeneration in the optic nerve at both 10.5 and 12 months [27].

**Figure 2:**
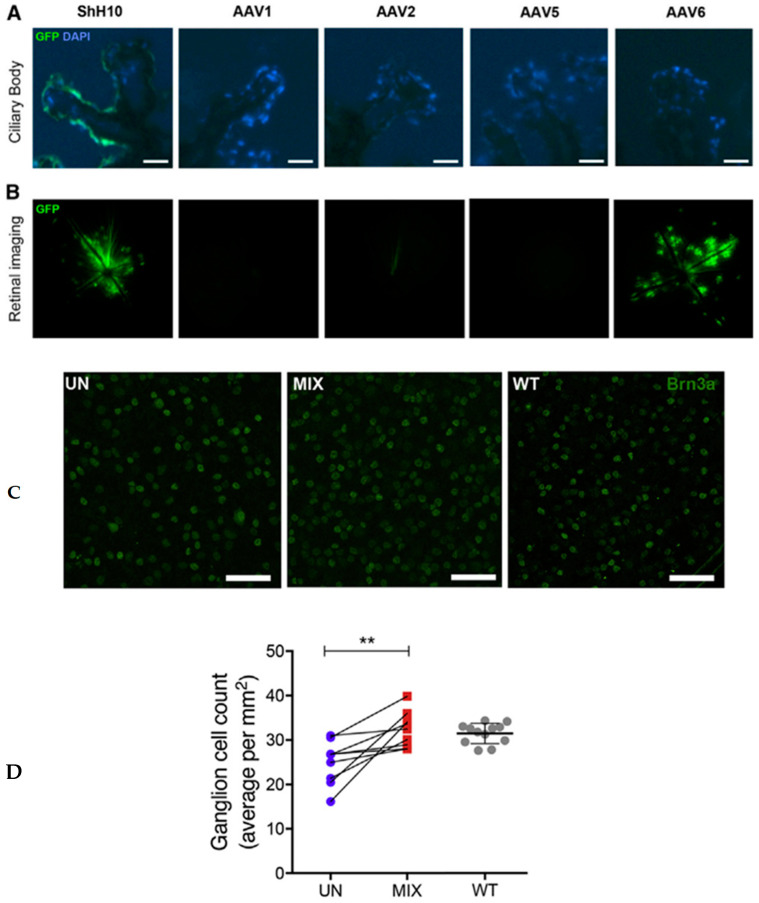
The ShH10 adeno-associated virus (AAV) serotype efficiently transduces the ciliary body epithelium following intravitreal injection. Four weeks after intravitreal administration of AAV encoding GFP driven by the CMV promoter, the ciliary body was evaluated using immunofluorescence techniques. Only the ShH10 serotype exhibited clear GFP expression in the ciliary body (**A**,**B**). Extending the evaluation to 7 weeks confirmed reduced ganglion cell loss in the treated group. Representative examples of retinal flatmount staining for Brn3a (**C**), along with ganglion cell quantification (**D**), showed that the mean Brn3a+ ganglion cell count in wild-type (WT) untreated retina was used as a reference. The quantification was based on the average of six fields per eye and expressed as mean ± SD per mm², collected from two independent experiments. Paired eyes were subsequently injected with either the ShH10-CMV-SaCas9-sgRNA B and E mix (MIX) or left untreated (UN) paired *t* test, n = 9 pairs. Mean ± SD is shown. Scale bars, 50 mm. ** *p* < 0.01 (Molecular Therapy, Jiahui Wu et al., 2020 [2]).

Another study evaluated the use of gene therapy to reduce intraocular pressure (IOP) by disrupting Aquaporin-1 in the ciliary body using the CRISPR-Cas9 system delivered via intravitreal injection. The authors demonstrated a targeted approach to gene therapy that selectively reduces IOP by inhibiting aqueous humor production in the ciliary body following a single intravitreal injection. In this study, a total of 2 × 10^10^ genome copies of various adeno-associated virus (AAV) serotypes (AAV encoding GFP driven by the CMV promoter) were injected into the vitreous cavity of one eye in each mouse. Wild-type C57BL/6J mice received a unilateral intravitreal injection of the combined vector mix, and 3 weeks later, the ciliary body was isolated. Four weeks after injection, (Figure 2A) expression in the ciliary body was assessed using immunofluorescence staining, and (Figure 2B) retinal transduction was examined through in vivo fundus fluorescence imaging. Among the tested serotypes, the ShH10 serotype was the only one to show strong GFP expression in the non-pigmented epithelium of the ciliary body at 4 weeks (Figure 2) [2]. The treatment resulted in an approximate 22% reduction in IOP, which would be clinically effective if applied in glaucoma therapy, as studies have shown that even a 25% reduction can help preserve vision. No statistically significant differences in IOP between eyes were identified at baseline, although greater variability was observed in IOP between different animals. Consequently, contralateral eyes were used as optimal controls, with data points displayed as linked eye pairs where appropriate. Extending the model to 7 weeks demonstrated that the treatment prevented retinal ganglion cell loss (Figure 2C,D), highlighting not only the IOP reduction but also cellular preservation—the ultimate goal of any glaucoma therapy.

### 2.3. Advances in Protection of RGCS by Retinal Glaucoma Cell Gene Therapy

Studies suggest that retinal ganglion cell (RGC) axons are affected in the early stages of glaucoma, independently of the soma. There is also evidence that the BAX-mediated axonal degeneration pathway plays a critical role. Gene delivery to RGCs is typically achieved through a single intravitreal injection at the sclero-corneal limbus. The viral vector used is the adeno-associated virus (AAV), a small, non-pathogenic virus (19–26 nm) that provides safe and long-term expression of the transgene. AAV has been employed as a vector to deliver the corrected version of the RPE65 gene, which is mutated in both animals and humans with Leber congenital amaurosis, a disease that leads to blindness. In clinical studies, the gene delivered to the subretinal space using a viral vector successfully restored vision in study participants [25].

In a study conducted in 2021, Ahmara et al [3]. demonstrated that the SIRT1 gene can prevent retinal ganglion cell (RGC) loss in models of optic neuropathy through either pharmacological activation or genetic overexpression. They investigated the neuroprotective potential of the SIRT1 gene by selectively targeting RGCs in a model of optic nerve injury (ONC). The study showed that adeno-associated virus (AAV)-mediated overexpression of SIRT1 in RGCs reduces RGC loss, thereby preserving visual function. Cohorts of C57Bl/6J mice were used, receiving intravitreal injections of either experimental or control AAVs, utilizing a ganglion cell-specific promoter or a constitutive promoter, followed by ONC induction. Visual function was assessed through the optokinetic response (OKR) 7 days post-ONC. Retinas and optic nerves were then collected to evaluate RGC survival using immunostaining techniques. The results indicated that selective RGC expression of SIRT1 provides targeted neuroprotection in an animal model with significant ganglion cell loss. Additionally, SIRT1 overexpression through AAV-mediated gene transduction suggests a selective neuroprotective effect on RGCs in the ONC model. In conclusion, the study provides valuable insights into the mechanisms of SIRT1-mediated neuroprotection in the context of compressive or traumatic optic neuropathy, positioning it as a promising therapeutic candidate for further testing in various types of optic neuropathies [3,24,27,28].

BCLXL gene therapy mitigates neuropathology in the DBA/2J mouse model of inherited glaucoma [27].

**Figure 3:**
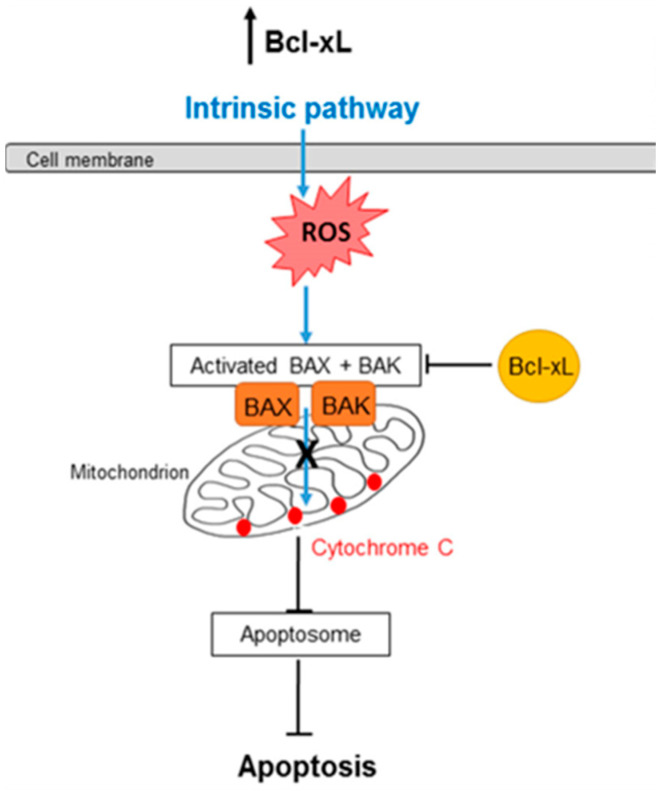
Gene therapy in animal models of glaucoma (adapted from [26,28,30]).

Bcl2l1 (hereafter referred to as BCLXL) is an anti-apoptotic member of the Bcl2 gene family and serves as the primary antagonist of BAX in central nervous system neurons. BCLXL is predominantly located in the mitochondria, where it inhibits BAX activation by preventing its accumulation on the outer mitochondrial membrane. Overexpression of BCLXL protects neurons from cell death following trophic factor withdrawal and ischemia-reperfusion injury [2,18,24,25,26]. In retinal ganglion cells (RGCs), increasing the intracellular concentration of BCLXL prevents the degeneration of somas and proximal axonal segments following axotomy. The DBA/2J mouse is a widely used glaucoma model that spontaneously and asynchronously develops elevated intraocular pressure (IOP) around 6 months of age, which persists until approximately 12 months. This prolonged elevation in IOP leads to RGC degeneration, making the DBA/2J mouse a valuable model for studying glaucomatous neurodegeneration. Results: Interestingly, deletion of the Bax gene provided protection only at 10.5 months, whereas removal of BIM, a protein crucial for BAX activation in RGCs, protected optic nerves (ONs) at both 10.5 and 12 months. This finding suggests that BCL2 family members may mediate axonal degeneration independently of BAX activity in RGCs. Gene therapy targeting RGCs to prevent BAX activation in a glaucoma model has not yet been performed, making this a clinically relevant approach that can also be tested in wild-type mice without the developmental defects associated with Bax deletion. BCLXL gene therapy resulted in better preservation of ONs in 12-month-old DBA/2J mice compared to those at 10.5 months. However, the increased levels of RGC-specific transcripts and the presence of the regeneration marker Gap43 indicate a certain degree of spontaneous regeneration occurring in these mice after ocular hypertension begins to subside, a process that starts between 11 and 12 months [6]. The effect of BCLXL on the regenerative potential of RGCs is a promising yet unexplored direction for future research. These findings suggest that BCLXL gene therapy preserves RGC anatomy and gene expression in a mouse model of glaucoma. To build on these results, future studies should investigate BCLXL gene therapy in a larger animal model of glaucoma (Figure 3, adapted from [25,26,27,29]).

### 2.4. Trabecular Meshwork Stem Cell Therapy

In glaucoma patients, a significant decrease in the number of trabecular meshwork (TM) cells leads to impaired aqueous humor outflow. Stem cells have been identified in the transition zone between the corneal endothelium and the non-filtering anterior portion of the TM. Utilizing these stem cells to repopulate the TM could potentially enhance drainage in glaucoma patients. Stem cell therapy holds promise for restoring TM function and protecting the optic nerve from further damage. Replacing damaged trabecular cells with healthy stem cells can restore the microenvironment of the filtering structures, leading to reparative proliferation and the restoration of physiological aqueous humor outflow, ultimately resulting in reduced intraocular pressure (IOP). Studies have successfully isolated and characterized stem cells from the human TM, demonstrating their ability to differentiate into functional trabecular cells, express specific TM markers, and exhibit phagocytic activity. Preliminary research on human tissue has shown the potential of induced pluripotent stem cells derived from the TM (iPSC-TM) to restore the homeostatic regulation of IOP in an ex vivo model using the human anterior segment. When cultured iPSC-TM cells were reintroduced into the anterior segment, they regained the ability to regulate aqueous humor outflow resistance in response to pressure changes [29,30,31,32,33,34,35,36].

To confirm the migratory capacity of trabecular meshwork stem cells (TMSCs) to the TM region and their survival after transplantation, various experiments were conducted in which human TMSCs and fibroblasts were labeled with a fluorescent membrane dye called DiO. These labeled cells were then injected into the anterior chamber of normal mice. The results demonstrated that the injected human TMSCs predominantly localized in the TM and remained viable within the tissue for at least 4 months. Within a week, some of the injected TMSCs began expressing the TM cell marker CHI3L1. In contrast, fibroblasts injected into the anterior chamber dispersed throughout the corneal endothelium, lens epithelium, iris, and TM but did not express the CHI3L1 marker. Minimal apoptosis was observed in the injected TM tissue, and intraocular pressure (IOP) did not increase during the experiment. Furthermore, no CD45-positive cells, indicative of an immune response, were detected after TMSC transplantation.

The conclusions drawn from these studies are as follows: TMSCs can be successfully isolated from the TM and expanded in vitro; TMSCs are capable of migrating to the TM region and differentiating into TM cells in vivo; and TMSC transplantation into the anterior chamber does not induce inflammation or trigger an autoimmune response that could lead to rejection. Additionally, another study showed that intravitreal injection of bone marrow-derived mesenchymal stem cells in a rat model of glaucoma resulted in increased overall RGC axon survival [26]. In conclusion, these cells may be valuable therapeutic agents for neurodegenerative diseases, as they have the ability to accumulate at injury sites, provide long-term neuroprotection after a single treatment, and protect the optic nerve from glaucomatous degeneration in experimental models.

## 3. Discussion

### 3.1. Future Directions

In summary, it is evident that multiple systems are available for gene delivery to relevant tissues in glaucoma gene therapy. Additionally, several therapeutic strategies can modulate the production and outflow of aqueous humor to regulate intraocular pressure (IOP) and protect retinal ganglion cells (RGCs) from apoptosis. As a result, the field of glaucoma gene therapy is well-positioned to offer significant advances in preventing blindness caused by this disease. However, further research is needed to optimize gene delivery methods for glaucoma treatment. A deeper understanding of the extracellular matrix, cytoskeletal remodeling, and cellular signaling events in the trabecular meshwork (TM) is crucial. Enhanced knowledge of the regulation and production of aqueous humor, as well as the factors released by the ciliary body that could influence TM function, is also necessary. Moreover, we need a clearer understanding of the cell death pathways activated in RGCs under various conditions, including elevated and non-elevated IOP. Developing animal models that accurately replicate changes in the TM and ciliary body, which lead to increased IOP, is critically important for testing new therapies. Furthermore, a better understanding of innate and antigen-specific immune responses to different vector systems is essential. It is particularly important to investigate the differences in these responses between rodents and primates to minimize adverse reactions. Identifying additional genes involved in glaucoma will provide valuable insights for developing new gene therapy strategies. Finally, it is essential to understand why the expression of ectopic genes delivered by different vectors ceases in various ocular cells and to identify promoters or other regulatory elements that can ensure long-term transgene expression [4]. Gene editing, which offers the potential for a one-time but permanent therapeutic modification, is an appealing approach, particularly because glaucoma is a chronic disease requiring lifelong management. Currently, progress remains in the preclinical stages, with strategies primarily focused on either modulating the trabecular meshwork to enhance aqueous humor outflow or providing neuroprotection to retinal ganglion cells.

### 3.2. Limitations of Gene Therapy

The primary limitation of gene therapy is its safety concerns, particularly regarding off-target effects. Modifying genes outside the targeted pathogenic site could result in the disruption of normal genes and the occurrence of unintended mutations, potentially leading to oncogenesis. There are also concerns about the potential of AAV vectors to trigger pro-oncogenic events, especially in the treatment of hematological diseases.

Additional issues related to viral vectors include toxicity to the patient, immune and inflammatory responses, and challenges in regulating gene expression. Since the mechanisms and pathways involved in the pathogenesis of glaucoma are not yet fully understood, precise genome editing requires further research.

Another limitation is the challenge of delivering a sufficient quantity of modified genes to the target tissues. Moreover, the high costs associated with developing experimental models present an additional hurdle. Ethical restrictions—most importantly, the ethical, legal, and social implications of germline editing—are always controversial and subject to ongoing debate.

## 4. Conclusions

There is huge potential when pharmaceutical/device technologies are combined with gene therapies to achieve superior efficacy and therapeutic outcomes for glaucoma patients. According to research, genomic therapy is a promising treatment for glaucoma and holds great potential for widespread clinical application.

We are on the brink of a new era in the treatment of glaucoma and other optic neuropathies. These emerging genetic technologies will move us beyond the current paradigm of simply prescribing eye drops to lower intraocular pressure. Stem cell therapy also offers hope for the future treatment of various eye conditions, including glaucoma.

Gene therapy could be utilized in two main ways in glaucoma: as a drug delivery system or as a foundation for developing new therapies and treatment endpoints targeting specific genetic mutations that cause the disease. In the future, patients could potentially begin therapy earlier in life and maintain a lower IOP to prevent glaucomatous progression and vision loss.

The transplantation of healthy stem cells into the anterior chamber has been shown to prevent pressure increases and maintain the density of retinal ganglion cells. Experiments using human trabecular meshwork stem cells have demonstrated their ability to migrate and survive in the trabecular region without inducing inflammation or triggering an autoimmune response. These findings underscore the potential of stem cell therapy in enhancing trabecular meshwork function for glaucoma treatment.

Consequently, genetic profiling can enable the customization of patient therapy, thereby providing a more stable and individualized approach to managing glaucomatous disease.

## Figures and Tables

**Table 1:** Types of tissues, vectors, routes, species, and the efficiency of these vectors.

Tissue or Cell Type	Vector	Route	Species	Efficiency
Trabecular Meshwork	Adenovirus	intracameral	rabbit	high
	Adeno-associated virus serotype 2,3,4	intracameral	monkey	high
	Herpes simplex Virus	intracameralar	rat, monkey	no transduction
	Lentivirus	tissue culture	human	no transduction
	Liposomes	intracameral	rodent, monkey	good
Ciliary Epithelium	Adenovirus	intracameral	oc-human,	high
	Adeno-associated virus	intracameral	rat, monkey	poor
	Herpes simplex virus	intracamerular	oc-human, rats	good
	Lentivirus	lens culture intracameral	rodent	unknown
	Liposomes	intracameral	monkey	unknown
Ciliary Muscle Cells	Adenovirus	tissue culture	human	unknown
	Adeno-associated virus			unknown
	Herpes simplex virus			good
	Lentivirus			unknown
	Liposomes			unknown
Retinal Ganglion Cells	Adenovirus	Intravitreal	rodent	poor
	Adeno-associated virus	Intravitreal	rat	high
	Herpes simplex virus	Intravitreal	rodent, monkey	good
	Lentivirus	Retrograde	rodent	variable
	Liposomes			unknown

**Table 2:** Summary of potential target genes and tissues for therapeutic use in glaucoma treatment.

Cell/Tissue Type	Target Gene	Predicted Effect
Trabecular meshwork	Cytoskeleton regulatory proteins	
Ciliary epithelium	Genes that regulate circadian rhythm of aqueous production beta-Adrenergic receptors Other genes modulating fluid production Neuropeptides	Reduce nighttime increases in aqueous production that lead to potentially damaging lOP levels Increasing the potential of ciliary body cells to respond to drugs that inhibit aqueous humor production Modulate TM and CM functions
Ciliary muscle cells	Gene X Metalloproteinases	Upregulation of prostaglandin synthesis
Retinal ganglion cells	Neurotrophin receptors (TrkB)\Neurotrophin genes BclX	Increase the potential for RGCs to respond to neurotrophins
Müller cells	GLAST Neurotrophins	Upregulate the endogenous glutamate transporter to enhance clearance of extracellular glutamate levels Provide a surrogate source of endorphins for RGCs (retinal ganglion cells).

**Table 3:** Gene variants associated with glaucoma.

Gene	Symbol	Gene Name	Genomic Location	Glaucoma Type	Populations
*ANP*	Atrial natriuretic polypeptide	1p36.2	108780	POAG	Caucasian
*APOE*	Apolipoprotein	19q13.2	107741 Neurodegenerative diseases, Alzheimer’s disease	NTG, POA	Japanese, Chinese, Tasmania, French
*CDH-1*	Cadherin 1	16q22.1	192090 Cell adhesion molecule	POAG	Chinese
*CYP1B1*	Cytochrome	P450, 1B1 2p22-p21	601771 Tryptophan metabolism	POAG	Indian, French, Spanish
*EDNRA*	Endothelin receptor	type A 4q31.2	131243 Calcium signaling pathway	NTG	Korean, Japanese
*GSTM1*	Glutathione S-transferase M1	1p13.3	138350 Glutathione metabolism	POAG	Arabs, Turkish, Estonian
*HSPA1A*	Heat shock 70 kDa protein 1A	6p21.3	140550 MAPK signaling pathway	POAG, NTG	Japanese
*IGF2*	Insulin-like growth factor2	11p15.5	147470	POAG	Chinese
*IL1α*	Interleukin-1 α	2q14	147760 MAPK pathway, apoptosis	POAG	Chinese
*IL1β*	Interleukin-1 beta	2q14	147720 Apoptosis, MAPK and Toll-like receptor signaling pathway	POAG	Chinese
*MTHFR*	Methylene-tetrahydrofolate reductase	1p36.3	607093 Folate biosynthesis, methane metabolism	NTG, POAG	Korean, Germany
*NOS3*	Nitric oxide synthase 3	7q36	163729 Arginine and proline metabolism, calcium and VEGF pathway	POAG with migraine history	Caucasian
*OCLM*	Oculomedin	1q31.1	604301	POAG	Japanese
*OLFM2 2*	Olfactomedin	19p13.2		POAG	Japanese
*OPA1*	Optic atrophy 1	3q28-q29	605290	NTG	Japanese, Caucasian
*P21*	P21	6p21.2	116899 p53 signaling pathway	POAG	Chinese
*PON1*	Paraoxonase 1	7q21.3	168820	NTG	Chinese
*TAP1*	ABC transporter, MHC, 1	6p21.3	170260 ABC transporters	POAG	Chinese
*PON1*	Paraoxonase	1 7q21.3	168820	NTG	Japanese
*TAP1*	ABC transporter, MHC, 1	6p21.3	170260 ABC transporters	POAG	Chinese
*TLR4*	Toll-like receptor 4	9q32-q33	603030 Toll-like receptor signaling pathway	NTG	Japanese
*TNFα*	Tumor necrosis factor alpha	6p21.3	191160 MAPK and Toll-like receptor pathway, apoptosis	POAG	Japanese, Chinese
*TP53*	Tumor protein 53	17p13.1	Genomic location MAPK and p53 pathway, apoptosis 191170	POAG	Chinese, Caucasian

**Table 4:** Current clinical trials for optic nerve disease.

Trials	Eyes Diagnostics	Location	Trial Summary	Phase	Trial Results
Dual intravitreal implantation of NT-501 encapsulated cells Therapy for glaucoma	Glaucoma	Stanford University	To determine the safety and efficacy of dual NT-501 CNTF encapsulated cell therapy (ECT) on visual impairment related to glaucoma	Phase II	Endpoints: visual fields, structure measurements, RNFL, and GCIPL
Study of NT-501 encapsulated cell therapy for glaucoma, neuroprotection, and vision restoration	Glaucoma	Stanford University	To determine the efficacy of NT-501 CNTF encapsulated cell therapy on visual impairment from glaucoma	Phase II	Endpoints: visuals fields, structure measurements, RNFL, and GCIPL
RESCUE and REVERSE long-term follow-up	Leber’s Congenital Optic Neuropathy	GenSight Biologics	To assess the long term and efficacy of G5010 and quality of life in subjects with LHON due to the G11778A mitochondrial mutation in patients five years post treatment	Phase III	Adverse events, BCVA and HVF
Safety study of an adeno-associated virus vector for gene therapy of Leber’s hereditary optic neuropathy	Leber’s Congenital Optic Neuropathy	National Eye Institute	To study the potentially toxic effects of scAAV2-P1ND4v2 in patients with LHON and G11778A	Phase I	Assessment of toxicity

## Data Availability

The datasets used and analyzed during the current study are available from the corresponding author upon reasonable request.
